# Lysosome and Cytoskeleton Pathways Are Robustly Enriched in the Blood of Septic Patients: A Meta-Analysis of Transcriptomic Data

**DOI:** 10.1155/2015/984825

**Published:** 2015-04-30

**Authors:** Jie Ma, Chuanxi Chen, Andreas S. Barth, Chris Cheadle, Xiangdong Guan, Li Gao

**Affiliations:** ^1^Division of Allergy & Clinical Immunology, Johns Hopkins University School of Medicine, Baltimore, MD 21224, USA; ^2^Department of Critical Care Medicine, The First Affiliated Hospital of Sun Yat-Sen University, China; ^3^Division of Cardiology, Johns Hopkins University School of Medicine, Baltimore, MD 21287, USA

## Abstract

*Background*. Sepsis is a leading cause of mortality in intensive care units worldwide. A better understanding of the blood systems response to sepsis should expedite the identification of biomarkers for early diagnosis and therapeutic interventions. *Methods*. We analyzed microarray studies whose data is available from the GEO repository and which were performed on the whole blood of septic patients and normal controls. *Results*. We identified 6 cohorts consisting of 450 individuals (sepsis = 323, control = 127) providing genome-wide messenger RNA (mRNA) expression data. Through meta-analysis we found the “Lysosome” and “Cytoskeleton” pathways were upregulated in human sepsis patients relative to controls, in addition to previously known signaling pathways (including MAPK, TLR). The key regulatory genes in the “Lysosome” pathway include lysosomal acid hydrolases (e.g., protease cathepsin A, D) as well as the major (LAMP1, 2) and minor (SORT1, LAPTM4B) membrane proteins. In contrast, pathways related to “Ribosome”, “Spliceosome” and “Cell adhesion molecules” were found to be downregulated, along with known pathways for immune dysfunction. Overall, our study revealed distinct mRNA activation profiles and protein-protein interaction networks in blood of human sepsis. *Conclusions*. Our findings suggest that aberrant mRNA expression in the lysosome and cytoskeleton pathways may play a pivotal role in the molecular pathobiology of human sepsis.

## 1. Introduction

Sepsis, a maladaptive response to infection, is a common and lethal syndrome. The hospital incidence of sepsis has been reported as high as 153/100,000 to 353/100,000 in both industrialized [1, 2] and also developing countries [3], with an increasing trend year by year [1–3]. In the United States, sepsis is one of the top ten leading causes of mortality [4]. Although adjusted in-hospital mortality has decreased gradually (2-3% per year) according to a recent report [1, 5], sepsis associated mortality remained high, from 50/100,000 to 75/100,000 [1, 6]. It was even higher when sepsis was accompanied by organ dysfunction, ranging from 23% to 58% with dysfunction of one organ [2, 3, 7] and increasing to 77.4% when three or more organs had failure [3]. Moreover, the respiratory system tended to be the most commonly infected site (49.3%) [1] and was associated with a high rate of organ failure (47.6–91.2%) [2, 8]. Elucidating the early pathogenesis of sepsis will likely decrease time to diagnosis and intervention, which would be expected to improve outcomes in critically ill patients.

The systemic inflammatory response syndrome (SIRS) contributes to multiple organ failure (multiple organ dysfunction syndrome) in sepsis and is accompanied by a first pro-inflammatory response and a following “immune paralysis” (compensatory anti-inflammatory response syndrome) responsible for secondary infections. This widespread and excessive reaction induces microthrombi formation, capillary obstruction, microcirculatory alterations, tissue edema by capillary leak, and neutrophil recruitment leading to multiple tissue damages, organ failures and finally to death [9]. There are three recognized stages of severe host response to pathogen with progressively increased mortality rates: sepsis, severe sepsis, and septic shock. Known pathways related to innate immune effectors, inflammatory mediators, and modulators of coagulation are particularly implicated in sepsis pathophysiology [10, 11]. The toll-like receptor (TLR) signaling pathway is not only relevant to immunosuppression but also to increasing neutrophil migration, vascular permeability, and proinflammatory cytokines in sepsis [12, 13]. The MAPK and NF-*κ*B signaling cascades, induced by the inflammatory mediators lipopolysaccharide (LPS) and TNFalpha in models of lung inflammation and sepsis, are known to promote endothelial cell hyperpermeability [14, 15]. In addition, inhibition of the NF-*κ*B signaling pathway in combination with antibiotics improved the survival rate in a sepsis model of cecal ligation and puncture (CLP) in rats, likely by reducing inflammation and attenuating vascular endothelial leakage [16–18]. Thus, activation of the procoagulant, proinflammatory, and proapoptotic pathways at the blood and tissue interface is one of the most prominent features of sepsis [19, 20].

Gene expression profiles enable systems-wide analysis of cellular processes and have provided novel insights on the molecular mechanisms underlying sepsis as will be discussed below. Genome-wide transcription profiling of human sepsis in host leukocytes revealed a consistent theme of activation of pathogen recognition pathways (including pathogen recognition receptors TLRs and CD14) and signal transduction pathways, which are processes essential for subsequent transcription of immune response genes [11]. In order to better understand the pathogenesis of sepsis at the transcriptional level and with the aim of translating emerging genomics data into new early diagnosis and effective treatment strategies, we used bioinformatics approaches to integrate the results from multiple microarray studies of sepsis. We selected datasets, which are freely available in the GEO (Gene Expression Omnibus, http://www.ncbi.nlm.nih.gov/geo/) data repository [22]. We chose datasets based on peripheral whole blood samples from patients with sepsis and from controls since blood is readily accessible and likely to reflect changes in disease related pathways.

## 2. Materials and Methods

### 2.1. Study Selection and Data Mining

**Figure 1:**
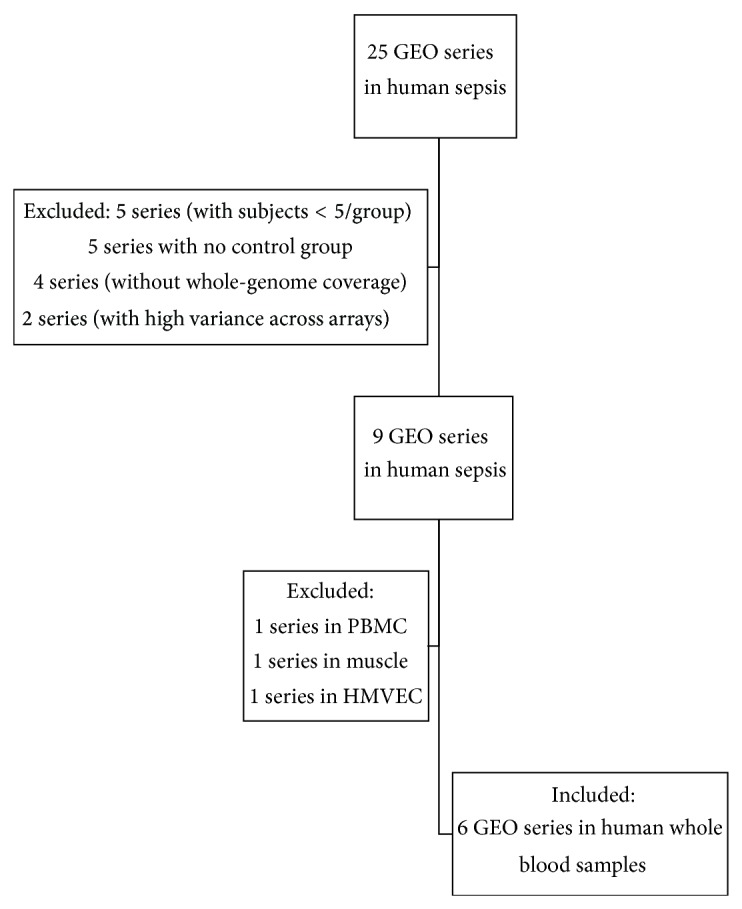
Selection of existing GEO series. A total of 25 GEO series in human were obtained using the key word “sepsis” and only 9 series met the criteria. We included 6 out of 9 series, which used whole blood samples, 3 series which used other tissue types (PBMC, muscle, and HMVEC) were excluded.

We searched GEO for experiments studying sepsis in human whole blood samples and identified 6 studies (GEO accession numbers: GSE30119, GSE8121, GSE9692, GSE28750, GSE13015 and GSE21802; Table 1(a)) fulfilling the following criteria: (1) sample size in each study should have *n* ≥ 5 in both the groups of sepsis patients and controls; (2) had whole genome-coverage (>20,000 genes) in each study; and (3) had low variance across arrays. All 6 studies were previously published [23–28] and were included in the meta-analysis (Figure 1). The 6 cohorts met the above selection criteria had included a total of 450 individuals (patients with sepsis = 323, controls = 127) providing genome-wide mRNA expression data (Table 1(a)). Patient characteristics, source of infection and time of data collecting of included studies were summarized in Table 1(b).

### 2.2. Identification of Common Pathways for Human Sepsis

All 6 studies were analyzed separately using the original Series Matrix File data format provided in GEO. Information on processing of samples including source of total RNA isolation, microarray hybridization protocol and data normalization methods were presented in Table S1 in Supplementary Material available online at http://dx.doi.org/10.1155/2015/984825. First, we used unpaired two-class Significance Analysis of Microarray (SAM) [29] to identify differentially expressed transcripts within each study (using log2-normalized microarray data and permutations = 200), which corresponds to ANOVA with correction for multiple testing. Differences in gene expression were regarded as statistically significant if a false discovery rate (FDR) of *q* < 0.1 was achieved, as previously described [30]. Second, we used the Kyoto Encyclopedia of Genes and Genomes (KEGG) (http://www.genome.jp/kegg/kegg2.html) [31] for pathway mapping. Briefly, results for each dataset from the SAM analysis were submitted to DAVID (http://david.abcc.ncifcrf.gov/) [32] under the functional annotation option specifying* Homo sapiens* as the species. Additionally, the threshold was modified as count = 1 and EASE-score = 10 to find every possible KEGG pathway [33]. Net expression of a pathway, defined as the number of upregulated transcripts minus downregulated transcripts expressed as percentage of the total number of genes within a KEGG pathway, was calculated to enable comparison of KEGG pathways representing a different number of genes, as described previously [33]. Third, rank scores were given to the top 10 up or downregulated KEGG pathways in each study (with rank scores assigned from 1 to 10, 10 represented the most significant pathway) and a top KEGG pathway was defined if it had the highest combined rank scores and most appearances across all studies. Finally, we visualized the net expression using Genesis software [34].

### 2.3. Identification of Candidate Genes in Top Common Pathways for Human Sepsis

We further analyzed each GEO dataset separately to identify individual differentially expressed genes. First, we extracted 122 genes in the lysosome and 215 genes in the cytoskeleton pathways in KEGG from the normalized dataset. Second, we estimated the mean difference between the average expressions of those genes per sample and used Student's *t*-test to determine statistically significant differential expression (both up- and downregulation) between sepsis patients and the controls, and this was done for all genes in both pathways for each dataset. Then we calculated the average *P* values for both pathways in each dataset (reported as −log(*P*) in Figure 3). Lastly, we reported significantly upregulated genes in sepsis patients satisfying a criteria of a *t*-test *P* value <0.01 and a fold change (FC) >1.5 (Table 3).

### 2.4. Network Analysis of Protein-Protein Interactions

Analysis of protein-protein interactions were conducted using the “Unified Human Interactome Database” (UniHI 7) [21], which contains more than 350,000 molecular interactions between genes, proteins, and drugs, as well as numerous other types of data such as gene expression and functional annotation. UniHI 7 constitutes one of the most comprehensive platforms for the query, visualization, and analysis of molecular networks in human. UniHI7 also allows the filtering of protein-protein interactions and provides “Phenotype Enrichment analysis,” thus enabling the construction of phenotype-specific networks. The display (Figure 5) was restricted to at least 3 literature references in PubMed. We also explored whether known drug targets and the phenotype of “Mortality and Aging” were enriched with network proteins.

## 3. Results

### 3.1. Pathways Enriched in Individual Studies

We first summarize the results found in the studies we examined and then describe the results of our meta-analysis in relation to these findings. The predominant pathogen was bacteria in 5 out of 6 published studies while one study (GSE21802) focused on influenza virus (with confirmed H1N1 infection). The primary sources of infection were blood and lung. Shanley et al. [23] found time-dependent, differential regulation of genes involved in multiple signaling pathways and gene networks primarily related to immunity and inflammation in pediatric patients with septic shock (*n* = 30), relative to controls (*n* = 15). Notably, interleukin-6, toll-like receptor, p38 MAP kinase, and NF-*κ*B signaling pathways were upregulated, on both day 1 and day 3, while antigen presentation, natural killer cell, and T cell receptor signaling pathways were downregulated. These findings were subsequently confirmed in an additional cohort of 30 children with septic shock and 14 separate normal controls [24]. Other studies aimed at detecting genes for early diagnosis of sepsis also demonstrated that genes directly involved in innate and early adaptive immune function (56%), cell cycling and white blood cell differentiation (32%), and extracellular matrix remodeling (7%), as well as immune modulation (5%), may serve as molecular biomarkers [25]. Another study of septicemic melioidosis, a severe infectious disease caused by the Gram-negative bacillus* Burkholderia pseudomallei*, identified a signature significantly enriched in genes coding for products involved in MHC class II antigen processing and presentation pathways [26]. The largest study was on children hospitalized with community-acquired* S. aureus* infection (*n* = 99) and an overall transcriptional signature of overexpression of innate immunity and hematopoiesis related genes as well as underexpression of genes related to adaptive immunity was identified [28]. In summary, there were critically important pathways representing an imbalance of pro- and anti-inflammation, immune dysfunction and modulation across the aforementioned studies.

### 3.2. Common Pathways Revealed in Septic Patients through Meta-Analysis

**Figure 2:**
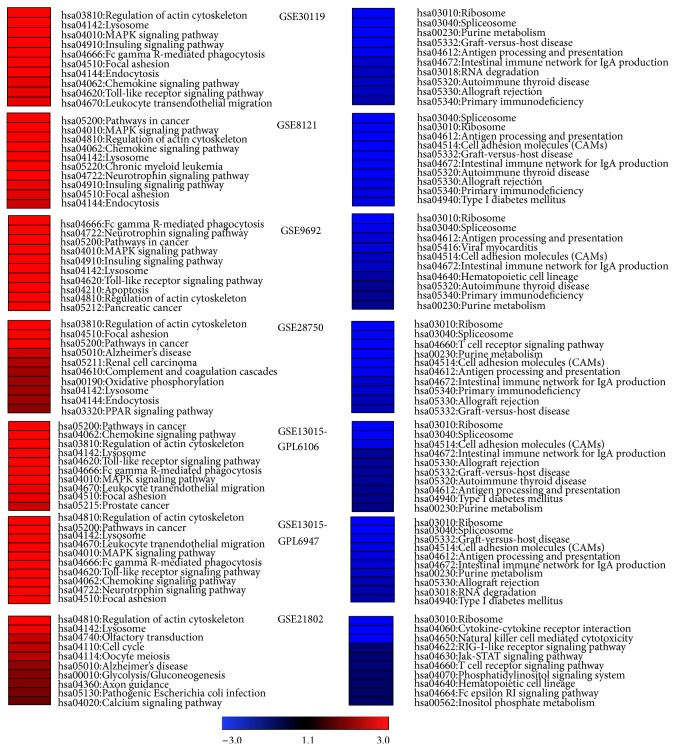
Top 10 up- and downregulated KEGG pathways in human sepsis. KEGG pathways were sorted ascending for upregulation and descending for downregulation by net expression and visualized in Genesis software. Here we only showed top ten pathways that had either the highest or the lowest net expression in each individual study. Blue color represents negative net expression and red color means positive net expression. The absolute net expression value is proportionate with color brightness, the brighter the color the higher the absolute value.

Out of a total of 429 KEGG pathways in the KEGG database, we identified 199 KEGG pathways with statistically significant regulation across the 6 studies in human sepsis we examined (listed in Supplementary Figure 1). The 199 KEGG pathways with statistically significant regulation displayed more consistent transcriptional patterns among the 6 studies with bacterial infection as compared to study GSE21802 that had virus infection. Sepsis is defined as systemic inflammatory response syndrome with infection and the pathogen includes bacteria, fugal, and virus. The heterogeneity we observed in the host response signatures for GSE21802 is likely influenced by the type of pathogen (e.g., virus versus bacteria). According to rank scores and frequency of appearance, we found robust upregulation of multiple KEGG pathways in whole blood samples of human sepsis across all studies examined, including “Lysosome,” “Regulation of actin cytoskeleton,” “Pathways in cancer,” “MAPKinase signaling,” “Fc gamma R-mediated phagocytosis,” “Chemokine signaling pathway,” “Toll-like receptor signaling,” “Neurotrophin signaling pathway,” “Insulin signaling pathway,” and “Focal adhesion” (Figure 2 and Table 2). In contrast, the top 4 downregulated pathways included “Ribosome,” “Spliceosome,” “Antigen processing and presentation,” and “cell adhesion molecules” (Table 2). Of note, in addition to well-known pathways involved in sepsis (e.g., MAPK signaling and Toll-like receptor signaling), our study identified novel signaling pathways “Lysosome” and “Regulation of actin cytoskeleton” as the two most significantly upregulated pathways across all 6 studies, suggesting that these 2 pathways might play a previously underrecognized role in human sepsis. More importantly, our study revealed an imbalance between protein synthesis (e.g., downregulation of “Ribosome” and “Spliceosome” pathways) and lysosome-mediated protein degradation (e.g., upregulation of “Lysosome” pathway), which may account for the multiple organ injury and failure in sepsis.

We recognize that the gene expression signature we identified through meta-analysis could be tissue- and disease stage-specific. We had restricted our selection criteria to include only studies performed on whole blood of human sepsis; this approach could reduce the heterogeneity in host response signatures due to various tissue types. However, the time of sample/data collection for each study varied, which could influence the precise patterns of temporal gene expression. This remained as one of the limitations of our study arising from the challenge of finding sufficient number of studies available in GEO for stratifying patients into more homogeneous populations.

### 3.3. Candidate Genes Differentially Expressed in Lysosome and Cytoskeleton Pathways

**Figure 3:**
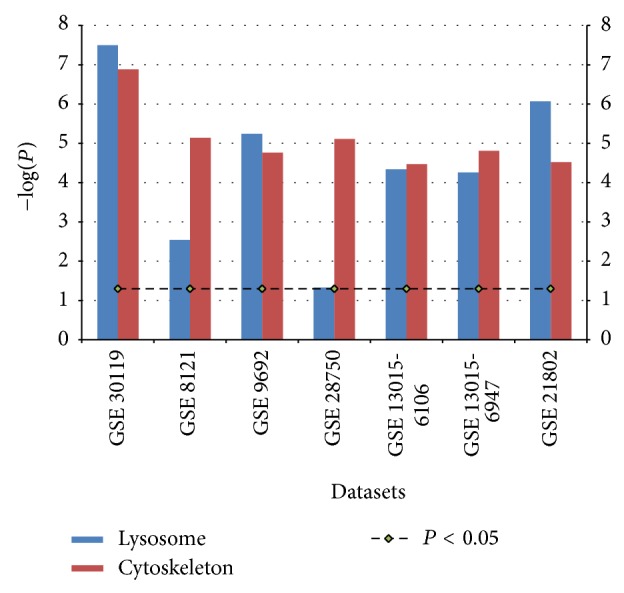
Lysosome and cytoskeleton pathways are enriched across multiple studies in blood of septic patients. This figure displays the −log *P* values (with *P* < 0.05 as cut-off) yielded from Student's *t*-test for the mean of average expression of genes in each pathway, comparing sepsis patients to the controls in each dataset, and only significantly up-regulated genes with either *P* value <0.01 or FDR >1.5 were included in the analysis.

**Figure 4:**
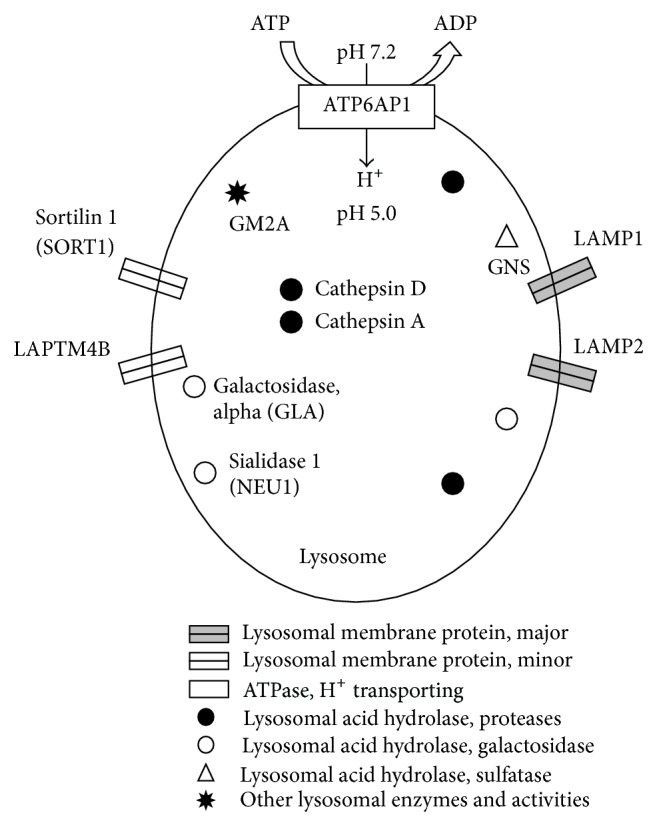
Target molecules in lysosome pathway dysregulated in human sepsis. Lysosome is a cytoplasmic organelle containing enzymes that break down biological polymers. Organization of the lysosome is depicted as dense spherical vacuole containing a variety of acid hydrolases (close circle: proteases; open circle: galactosidase; open triangle: sulfatase) that are active at the acidic pH maintained within the lysosome. Furthermore, both the major (double rectangle boxes in grey) and minor (double rectangle boxes) lysosomal membrane proteins are involved. ATPase (H^+^ transporting) is displayed as open rectangle and “other lysosomal enzymes and activities” are displayed as close star.

Given that lysosome and cytoskeleton were novel pathways for sepsis and among the most significantly upregulated in our study, we further examined individual genes dysregulated in these 2 pathways, the relation/interaction among them and their utilities as potential biomarkers for sepsis. We identified significantly upregulated genes in both the lysosome (a) and cytoskeleton (b) pathways across multiple studies by comparing the mean of average expression of each gene in the group of sepsis patients relative to the controls. Both the −log *P* values and fold changes are displayed for the top 15 genes most significantly upregulated across studies (Table 3). Interestingly, genes encoding multiple lysosomal acid hydrolases including serine protease cathepsin A (CTSA) and aspartic proteases cathepsin D (CTSD), galactosidase, alpha (GLA), and sulfatase (GNS) as well as the major (LAMP1 and LAMP2) and minor (SORT1 and LAPTM4B) lysosomal membrane proteins are among the top dysregulated molecules. Additionally, vacuolar ATPase (ATP6AP1), which is responsible for proton transport, was also activated leading to acidification, which then activates hydrolytic enzymes [35]. Overall, these findings suggested that the lysosomal activities were increased in human sepsis (Figures 3 and 4). According to our search of the PubMatrix database (http://pubmatrix.grc.nia.nih.gov/), 9 out of the 15 upregulated genes in the lysosome pathway (including* GLA*,* CD63*,* GNS*, and* LAMP2*) and 7 in the cytoskeleton pathway (including* CD14*, integrins, and* MAPK1*) have been reported in the literature associated with search terms “sepsis,” “severe sepsis,” or “septic shock” (Table 3).

As a proof of concept, we further evaluated whether these significantly differentially expressed transcripts or their coding protein products previously had been utilized as biomarkers for sepsis. Currently the measurement of plasma C-reactive protein (CRP) and procalcitonin (PCT) has been utilized in diagnosing and monitoring neonatal sepsis, severe sepsis and septic shock, at the onset and in the course of the disease [36]. Commercial methods for the automated measurement of the soluble CD14 subtype presepsin (sCD14-ST) and lipopolysaccharide binding protein (LBP), the two old biomarkers, have been proposed over the past years to manage critically ill newborns with acute inflammation and sepsis. Presepsin levels in blood could also predict bacteremia in patients with SIRS in the Emergency Department [37]. Neutrophil activation marker CD63 was upregulated in patients with sepsis, suggesting that circulating neutrophils are fully activated in sepsis [38]. Another study which evaluated leukocyte activation in sepsis showed that cell surface expression of all activation markers (CD11b, ICAM-1, CD66b, CD63, and CD64) was increased on both neutrophils and monocytes from sepsis patients compared to healthy controls [39]. However, many genes in these two pathways have remained largely unexplored, and our results provide evidence for their involvement in the transcriptomic changes in human sepsis. We further evaluated all upregulated genes in these two pathways in each study, comparing sepsis patients to the controls in each dataset, and found that the lysosome and cytoskeleton pathways are overall dysregulated across all studies in blood of septic patients (Figure 3).

### 3.4. Network Analysis for Enrichment of Mortality Phenotype and Drug Targets

The network-based approaches can provide the most unbiased analysis of high-throughput omics data (e.g., gene expression array), a better understanding of human diseases on the systems-level [40], and help network-related drug design. Among different molecular networks, protein-protein interaction (PPI) networks have emerged as an important resource for understanding data from gene expression or proteomics experiments. Three consecutive steps are typically required to perform PPI network analysis [40]. The first step is to identify genes (e.g., differentially expressed genes) or proteins of interest. In the second step, these inputs (also known as “seed proteins”) are used to search and retrieve binary interactions from a curated PPI database. The third step is network analysis such as the topology analysis that considers the whole network structure to search for important nodes (hubs) that are useful as biomarkers or therapeutic targets.

**Figure 5:**
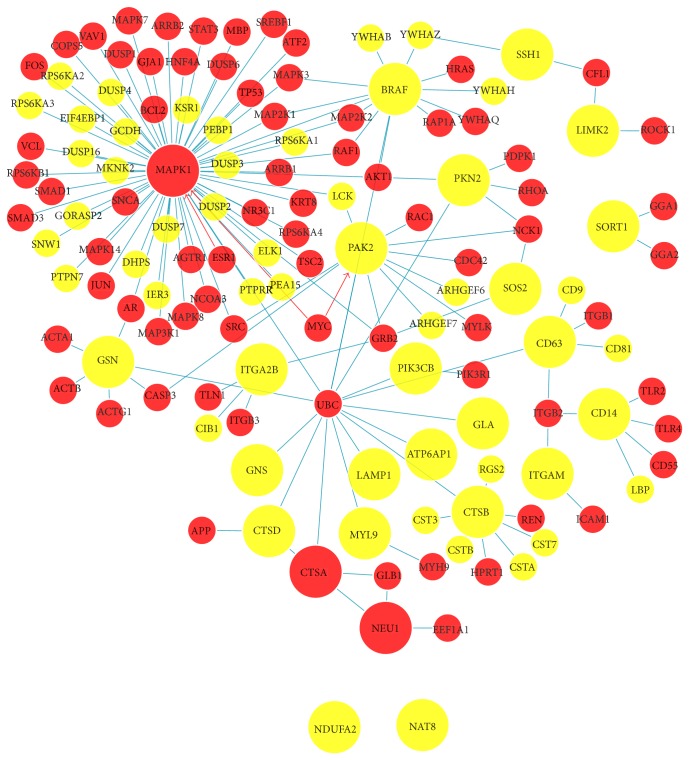
Graphical representations of protein interactions enriched for mortality phenotype. We queried the Unified Human Interactome database (UniHI 7) [21] with a list of top 15 transcripts from each of the two pathways. The display was restricted to those interactions (yellow proteins) supported by at least three PubMed references. Molecular network analysis revealed that 22 out of 30 transcripts formed a network with multiple interconnected nodes (hubs) between top genes in both pathways. Importantly, many of the predicted interaction partners (red proteins), including 3 query proteins (MAPK1, CTSA, and NEU1), were associated with the mortality phenotype.

We hypothesize that the changes in steady-state mRNA levels also have a profound effect on the expressed proteome. We are particularly interested in defining a set of proteins that mediate the cross-talk between lysosome and cytoskeleton pathways. Therefore, we searched for protein-protein interaction partners for the top 15 most significantly upregulated transcripts in each pathway for molecular network analysis using the “Unified Human Interactome Database” (UniHI 7) which has enhanced features allowing “Phenotype Enrichment analysis” that enables the construction of mortality-specific networks. We identified 21 highly interconnected nodes “hubs” linking the 30 most significantly upregulated transcripts in both pathways (Figure 5 and Supplementary Figure 2). The resulting protein-protein interaction network included 10 upregulated transcripts (*SORT1*,* CTSA*,* CTSB*,* CTSD*,* CD63*,* GNS*,* GLA*,* NEU1*,* ATP6AP1*, and* LAMP1*) in the lysosome pathway and 12 upregulated transcripts (*ITGAM*,* LIMK2*,* PIK3CB*,* SOS2*,* GSN*,* SSH1*,* PAK2*,* CD14*,* ITGA2B*,* BRAF*,* MYL9*, and* MAPK1*) in the cytoskeleton pathway (Figure 5). Importantly, Phenotype Enrichment analysis revealed that a subset of the transcripts (including* MAPK1*,* CTSA*, and* NEU1* among the top transcripts) in this highly coordinated network was enriched for mortality.

Prior evidence was found supporting the role of MAPK signaling pathway molecule MAPK1 in regulating cytoskeletal rearrangements and sepsis mortality. The MAPK signaling pathway is believed to play an important role in mediating proinflammatory responses and is drug targets for treatments of sepsis. MAPK1 (ERK2) and MAPK3 (ERK1) are the 2 MAPKs, which play an important role in the MAPK/ERK cascade. Depending on the cellular context, the MAPK/ERK cascade mediates diverse biological functions such as cell growth, adhesion, survival, and differentiation through the regulation of transcription, translation, and cytoskeletal rearrangements. In CLP-treated septic mice, p38 MAPK inhibitor (SB203580) significantly inhibited high mobility group box 1 (HMGB-1) release and increased survival rate [41], HMGB-1 is an endogenous ligand for TLR4, in addition to LPS, and an important mediator of sepsis-associated death in experimental studies [42]. In a human model of endotoxemia, the oral administration of a new p38 MAPK inhibitor reduced cytokine production and leukocyte responses. Additionally, many of the interacting molecules in this network were drug targets. However, the most significantly upregulated transcripts identified in our study had not been directly targeted by any drug treatments (Figure 2 in Supplementary Material), suggesting more studies are warranted targeting these dysregulated molecules as novel therapeutic targets for sepsis.

## 4. Discussion

Sepsis and septic shock are a major cause of mortality in intensive care units. The pathophysiology of sepsis involves highly complex interactions between invading microorganisms, the innate and adaptive immune systems of the host, and multiple downstream events leading to organ dysfunction [43]. Despite major advances in our understanding of the pathophysiology of sepsis, we still lack the tools and indicators for early diagnosis. Gene profiling of human peripheral blood cells has been successfully employed for biomarker discovery in various lung diseases including asthma [44], pulmonary hypertension [45], and COPD [46]. To investigate whether peripheral blood can serve as an accessible surrogate tissue outside of the lung for noninvasive discovery of molecular biomarkers for sepsis, we performed a meta-analysis of gene expression microarray data from human whole blood studies of sepsis, to identify specific pathways most implicated in sepsis. Our data provides evidence that gene expression profiling using whole blood can help elucidate novel pathophysiological mechanisms that may play a critical role in sepsis.

A complex network of biological mediators underlies the clinical syndrome of sepsis. Biomarker discovery utilizing systems-level approaches promises to transform sepsis from a physiologic syndrome to a group of distinct biochemical disorders. Furthermore, greater understanding of the complex network of immune, inflammation, coagulation, intermediary metabolism and other specific mediators of sepsis may allow the development of rational and novel therapies [47, 48]. Most of the distinct molecules proposed as useful biological markers of sepsis were implicated in the immunoinflammatory process common to sepsis including the production of proinflammatory cytokines, adhesion molecules, vasoactive mediators and reactive oxygen species [49]. More systematic investigations are needed to unravel the nature of the dysregulated molecular interaction networks during sepsis.

A systematic review [11] of 12 genomic studies that examined the host response of circulating leukocytes to human sepsis showed that there was an immediate activation of pathogen recognition receptors, such as TLRs and CD14, accompanied by an increase in the activities of signal transduction cascades (included NF-*κ*B, MAPK, JAK and STAT pathways), a process essential for subsequent transcription of immune response genes. In contrast, sepsis related inflammatory changes are highly variable on a transcriptional level. Established inflammatory markers, such as TNF-*α*, IL-1 or IL-10, did not show any consistent pattern in their gene-expression across cohorts [11]. In our meta-analysis, a consistent pattern of over-expression was seen for pathways well known to be involved in sepsis (e.g., TLR and MAPK signaling) as well as for the lysosome and cytoskeleton pathways, which had not been recognized in individual studies of sepsis reported previously. Additionally, the “Ribosome,” “Spliceosome,” and “Cell adhesion molecules (CAMs)” pathways were downregulated across most of the datasets examined, which were also novel findings revealed through the meta-analysis. These findings suggest overall there was an imbalance between the rate of protein synthesis and protein degradation or proteolysis, which can lead to protein wasting and multiple organ system dysfunction during sepsis [50]. The manifestation in skeletal muscle is a loss of skeletal muscle mass producing diminished muscle strength [51–53]. Sepsis induces the loss of muscle proteins by impairing skeletal muscle protein synthesis through an inhibition of messenger RNA (mRNA) translation by the ribosome [54]. Muscle weakness in septic patients contributes to a continued dependence on mechanical respirators, an increased risk of pneumonia, and the complications associated with reduced ability for ambulation. These clinical complications prolong hospitalization and convalescence, thereby increasing health-care costs [55].

Our meta-analysis included 6 studies utilizing whole blood samples. In addition to activation of pathways related to inflammation, signaling, and immunity, which were common pathways across all 6 studies examined, we found robust upregulation of the “Lysosome” and “Regulation of actin cytoskeleton” pathways in all these studies. Lysosomes are ubiquitous membrane-bound intracellular organelles containing more than 40 hydrolases in an acidic environment (pH of about 5, Figure 4). They are central for degradation and recycling of macromolecules delivered by endocytosis, phagocytosis, and autophagy. Of note, both the “Phagocytosis” and “Endocytosis” pathways were upregulated in our study (Table 2), along with the “Lysosome” pathway. Further, lysosomes contribute to cellular homeostasis by way of their involvement in secretion, plasma membrane repair, cell signaling and energy metabolism, which are related to health and diseases [56]. The severe consequences of inadequate lysosome function are seen in genetically inherited lysosomal storage diseases (LSD). The primary cause is deficiency of an acidic hydrolase, while there are also conditions arising from defects in lysosomal membrane proteins that fail to transport an essential enzyme [57]. The function of lysosomes is critically dependent on soluble lysosomal hydrolases (e.g., cathepsins) as well as lysosomal membrane proteins (e.g., lysosome-associated membrane proteins) [58]. In contrast to the rather simplified view of lysosomes as simply sites for disposal of macromolecules that have been designated for degradation, lysosomes are now recognized as advanced organelles involved in many cellular processes and are considered crucial regulators of cell homeostasis. There is increasing evidence indicating that lysosomes are involved in more widespread diseases, such as cancer, Alzheimer's disease [59], and amyotrophic lateral sclerosis [60]. Moreover, due to the essential role of lysosomes in autophagy, lysosomal dysfunction impairs this process, thereby contributing to disease [61]. In our study, we found that overall lysosomal pathway was activated in human sepsis with multiple lysosomal hydrolases and membrane proteins involved (Figure 4), suggesting the implication of lysosome system dysfunction to the pathogenesis of sepsis. It has been demonstrated recently in rats that a hyper consumption and subsequent reformation of the lysosome to meet the increased demand for autophagosome cleaning, a cellular defense system to prevent harmful effects, may be involved in cardioprotection against LPS-induced septic insults [62]. Another study [63] also demonstrated that 90% of intracellular bacteria were killed within 3 hours after Human Brain Microvascular Endothelial Cells were infected with* S. pneumoniae* and inhibition of lysosomal function resulted in a great increase of intracellular bacteria survival suggesting that the bacteria was destroyed in the lysosome.* In vivo*, it was also proved that lysosome played a protective role against sepsis. The levels of autolysosome were significantly increased in the liver of septic mice induced by CLP and inhibition of lysosomal function caused higher level of aspartate transaminase and reduced mice survival [64].

The neutrophil is a key contributor to the innate immune response and plays an important role in the pathogenesis of sepsis-induced multiple organ dysfunction. Specific inflammatory stimuli such as LPS, cytokines, chemokines and the adhesion molecules can lead to upregulation of neutrophil activity; drive pulmonary neutrophil transendothelial migration (TEM), a paradigm of rolling/adhering/diapedesis, and neutrophil-mediated inflammation leading to multiple tissue damages, organ failures and finally to death [65]. Notably, the “Leukocyte transendothelial migration” pathway was significantly upregulated in our study (Table 2), suggesting neutrophil activation in blood of human sepsis. The antimicrobial activity of the neutrophil is nonselective, so the activity of neutrophil-mediated inflammation must be regulated to prevent tissue injury. Pathogens are degraded into fragments and these nonhost peptides processed via the endosomal pathway become antigens that, in turn, are presented to T-cells. The antigen presenting nature of neutrophils during inflammation is supported by evidence that neutrophils contain reserves of cathepsin B and D, lysosomal proteases necessary for antigen presentation [66]. Cathepsins are a group of lysosomal proteases that have a key role in cellular protein turnover. The main function of cathepsins is protein recycling within the lysosome but they are also known to be involved in a range of other physiological, as well as pathological processes, including sepsis. In addition to the serine proteases cathepsins A and G, and ubiquitous aspartic proteases cathepsins D and E (only present in endosomes), the most important are cysteine cathepsins. In humans, there are 11 cysteine cathepsins known at the sequence level including cathepsins B, C (dipeptidyl peptidase I, DPPI) [67]. Cathepsin C plays an important role in bacterial killing and immune regulation, the activities of three serine proteases (neutrophil elastase, cathepsin G, and proteinase 3); all require cathepsin C activity for processing and maturity. It was found in mice that restricting cathepsin C activity could negatively affect neutrophil bactericidal killing activity [68]. In our study, both cathepsins A and D were among the top dysregulated molecules in the lysosome pathway in human sepsis. While the pathogenesis of sepsis is increasingly well understood, the involvement of the lysosomal pathway in neutrophil-mediated inflammatory diseases remains incompletely characterized, as well as its dynamic interaction with other major pathways (e.g., cytoskeleton and MAP-Kinase signaling).

During sepsis-causing infections, the vasculature is profoundly altered by the combination of microbial virulence factors and proinflammatory mediators released from activated blood cells that gain access to surrounding tissue by crossing the leaky endothelial boundary. Severe endothelial dysfunction then results from the loss of homeostatic function of the microvascular endothelium and contributes to hypoxic injury of multiple organs. The cytoskeleton is a necessarily dynamic structure for cells which plays a crucial role in regulating permeability [69] of cells under physiologic and pathophysiologic conditions. The cytoskeleton pathway is a known modulator of endothelial barrier function and microvascular permeability [70]. Recent compelling evidence suggests that the breakdown in blood/endothelial barrier function plays a crucial role in the pathogenesis of sepsis and organ dysfunction [71] and could be a new target for sepsis therapeutics [72]. It is well known that an inflammatory stimulus leads to development of capillary leak and tissue edema due to cytoskeleton rearrangement, which is a characteristic feature of sepsis and its development is one of the causes of organ failure in that syndrome [9]. Capillary leak may also result from endothelial cells injury and death [73]. Nonmuscle myosin light-chain kinase (MYLK) mediates increased lung vascular endothelial permeability in LPS-induced lung inflammatory injury and it was demonstrated that MYLK was also essential for neutrophil transmigration [74]. We have reported previously that MYLK genetic variants conferred risk for severe sepsis and sepsis-associated acute lung injury [75, 76]. Thus, investigation of the interaction among these key regulatory pathways is challenging and could result in significant protein remodeling in the blood and could have a profound impact on inflammation, coagulation and morbidity in sepsis.

The source of variation in gene expression studies in sepsis includes heterogeneity of the causes and microbiology of sepsis; variability in time from onset of sepsis to time of blood draw; and differences between tissues and variability in sample size [77]. There are limitations in our study, which depended on the particular available datasets. In particular, sepsis-associated organ dysfunction is the main cause of high mortality and therefore has been given particular attention [1–3, 7]. However, no dataset in GEO related to sepsis-associated organ dysfunction met our criteria for inclusion in our meta-analysis, so we were unable to examine target genes specific for severity and outcome of sepsis. The network analysis revealed complex interaction among target molecules and cross-talk between lysosome and cytoskeleton pathways. Most importantly, this highly coordinated interaction network was enriched for the mortality phenotype and drug targets and provided additional molecular targets for therapeutic interventions for sepsis. Validation of identified novel targets as potential biomarkers for an early diagnosis of sepsis utilizing standardized biomarker methodologies, and integration of biomarkers into clinical studies (in particular, early phase studies) are warranted.

## 5. Conclusions

Our meta-analysis has gained novel insights on sepsis pathogenesis and confirmed systematic changes in lysosome and cytoskeleton pathway genes in sepsis. Such insights into the molecular biology of sepsis may ultimately lead to effective antisepsis therapeutics. Our findings also suggest that blood samples may provide a means for relatively noninvasive detection of sepsis, which may aid in the development of tests providing early diagnosis and thereby better treatment outcomes in sepsis.

## Supplementary Material

The Supplementary Material includes one supplementary table and two additional supplementary figures that are complementary to the results reported in the final paper. These data are peer-reviewed and are subject to the same criteria as data in the main article. Each of the two figures is displayed with their legends placed below each corresponding figure

## Figures and Tables

**(a):** 

GEO accession	Comparison groups	Probe ID	Publication (PMID)
GSE30119	Healthy (*n* = 44) versus sepsis (*n* = 99)	48802	22496797

GSE8121	Nonseptic (*n* = 15) versus septic shock_day 1 (*n* = 30) versus septic shock_day 3 (*n* = 30)	54675	17932561

GSE9692	Normal (*n* = 15) versus septic shock (*n* = 30)	54675	18460642

GSE28750	Healthy (*n* = 20) versus Sepsis (*n* = 10) versus post-surgical (*n* = 11)	54675	21682927

GSE13015^∗^	Platform6106: nonsepsis (*n* = 19) versus septicemic melioidosis (*n* = 24) versus sepsis_other infection (*n* = 24)	48687	19903332
	Platform6947: nonsepsis (*n* = 10) versus melioidosis (*n* = 16) versus sepsis_other infection (*n* = 13)	48803	19903332

GSE21802	Healthy (*n* = 4) versus sepsis without MV (*n* = 16) versus sepsis with MV (*n* = 20)	48701	20840779

**(b):** 

GEO accession	Patients characteristics	Source of infection	Time of data collection
GSE30119	Age >10 and <18 year-old Septic pediatrics	Blood and marrow or arthritis (*n* = 56) Lung (*n* = 11) Skin (*n* = 10) Only 77 patients had identified positive infecting organism	Different days during hospitalization ≤3 days (*n* = 34) 3–7 days (*n* = 43) ≥7 days (*n* = 22)

GSE8121	Age <10 year-old Septic shock pediatrics	Blood (15) Lung (3) Urine (1) Retropharyngeal abscess (1) Only 20 patients had identified positive infecting organism	(1) Within 24 h of admission to ICU (day 1) (2) 48 h after day 1 (day 3)

GSE9692	Age <10 year-old Septic shock pediatrics	Blood (10) Lung (1) Cerebral spinal fluid (1) Other (2) Retropharyngeal abscess (1) Only 15 patients had identified positive infecting organism	Within 24 h of admission to ICU

GSE28750	Age >18 year-old BMI < 40 Septic patients	All blood	Septic patients: within 24 h of admission Post-surgical patients: within 24 h following surgery

GSE13015^∗^	Age >18 year-old Septic patients	All blood	Within 7 days of diagnose

GSE21802	Age >18 and <65 year-old Septic patients with confirmed H1N1 infection	Lung (*n* = 19)	Early period (before day 9 in the course of the disease) Late period (from day 9 in the course of the disease)

^∗^GSE13015 was a published study using 2 different microarray platforms and in different patients, therefore was considered as two separate studies in the statistical analysis.

**Table 2:** Whole blood transcriptional profiles identified top up- and downregulated pathways in human sepsis.

Upregulated pathways	Rank scores^∗^	Downregulated pathways	Rank scores^∗^
Lysosome	47	Ribosome	69
Regulation of actin cytoskeleton	46	Spliceosome	55
Pathways in cancer	45	Antigen processing and presentation	35
MAPK signaling pathway	34	Cell adhesion molecules	34
Fc gamma R-mediated phagocytosis	26	Intestinal immune network for IgA production	31
Chemokine signaling pathway	23	Graft-versus-host disease	28
Toll-like receptor signaling	17	Purine metabolism	21
Neurotrophin signaling pathway	16	Autoimmune thyroid disease	15
Insulin signaling pathway	16	Allograft rejection	14
Focal adhesion	14	T cell receptor signaling pathway	13
Leukocyte transendothelial migration	12	Cytokine-cytokine receptor interaction	9
Alzheimer's disease	12	Primary immunodeficiency	8
Endocytosis	8	Natural killer cell mediated cytotoxicity	8
Olfactory transduction	8	Viral myocarditis	7
Cell cycle	7	Hematopoietic cell lineage	7
Renal cell carcinoma	6	RIG-I-like receptor signaling pathway	7
Oocyte meiosis	6	RNA degradation	6
Chronic myeloid leukemia	5	Jak-STAT signaling pathway	6
Complement and coagulation cascades	5	Type I diabetes melitus	4
Oxidative phosphorylation	4	Phosohatidylinositol signaling system	4
		Fc epsilon RI signaling pathway	2
		Inositol phosphate metabolism	1

^∗^Pathways were listed according to rank scores (from high to low). Rank scores were given to the top 10 up- or downregulated KEGG pathways in each study (with rank scores assigned from 1 to 10, 10 represented the most significant pathway) and a top KEGG pathway was defined if it had the highest combined rank scores and most appearances across all studies.

**Table:** (a) Lysosome pathway

Gene Symbol	Gene Name	#1	#2	#3	#4	#5	#6	#7	PubMatrix^∗^
*SORT1 *	Sortilin 1	2.16 (13)	4.05 (9.8)	4.48 (10.5)	5.19 (8.4)	4.86 (4.4)	7.82 (2.3)	—	0
*CTSD *	Cathepsin D	1.86 (10.9)	2.57 (7)	2.36 (6)	2.1 (8)	2.75 (8.8)	—	5.94 (5.9)	3
*SLC11A1 *	Solute carrier family 11 (proton-coupled divalent metal ion transporter), member 1	3.69 (5.6)	2.62 (6.5)	3.98 (8.4)	2.4 (5)	3.31 (5.3)	3.85 (4.7)	—	4
*CD63 *	CD63 molecule	2.02 (5.9)	3.35 (8.4)	3 (10.5)	2.84 (8.7)	2.82 (6.1)	—	—	38
*GNS *	Glucosamine (N-acetyl)-6-sulfatase	—	3.17 (5.9)	3.91 (8.3)	2.76 (6.3)	1.85 (5.8)	—	2.77 (4.4)	10
*GM2A *	GM2 ganglioside activator	—	1.85 (4.2)	2.55 (6.5)	2.42 (7.6)	3.11 (5)	6.17 (3.7)	—	0
*LAMP2 *	Lysosomal-associated membrane protein 2	—	—	1.79 (2.2)	1.63 (3.7)	1.56 (6)	2.02 (3.9)	3.66 (2.7)	6
*LAPTM4B *	Lysosomal protein transmembrane 4 beta	1.76 (7)	—	1.54 (3.4)	1.82 (2)	2.31 (3.3)	2.07 (3.8)	—	0
*CTSA *	Cathepsin A	1.98 (13.6)	2.13 (5.3)	2.26 (4.8)	1.81 (4.3)	—	—	—	4
*GLA *	galactosidase, alpha	—	1.63 (5.2)	1.65 (4.3)	1.52 (7.4)	1.68 (6.5)	—	1.59 (2.6)	95
*NEU1 *	sialidase 1 (lysosomal sialidase)	—	1.67 (4.1)	1.65 (3.6)	—	1.87 (9.3)	—	1.93 (2.1)	0
*CTSB *	cathepsin B	—	2.41 (6.2)	2.05 (4)	—	—	1.84 (5.1)	1.78 (3.3)	2
*ATP6AP1 *	ATPase, H+ transporting, lysosomal accessory protein 1	—	—	1.59 (3.8)	—	1.67 (4.4)	2.12 (4.3)	1.57 (4.1)	0
*LAMP1 *	lysosomal-associated membrane protein 1	—	1.55 (2.1)	1.62 (3.2)	—	—	1.68 (5.3)	—	4
*AP3B2 *	adaptor-related protein complex 3, beta 2 subunit	—	—	1.6 (2.1)	2.32 (4.6)	266.89 (3.8)	—	—	0

**Table:** (b) Cytoskeleton pathway

Gene Symbol	Gene Name	#1	#2	#3	#4	#5	#6	#7	PubMatrix^∗^
*ITGAM *	integrin, alpha M (complement component 3 receptor 3 subunit)	1.72 (7.98)	2.82 (6.41)	2.88 (9.42)	3.19 (8.44)	3.16 (9.19)	4.36 (6.19)	4.01 (3.99)	356
*LIMK2 *	LIM domain kinase 2	1.69 (3.39)	2.83 (5.24)	4.3 (9.27)	2.08 (4.79)	5.72 (8.34)	11.72 (4.1)	—	0
*PDGFC *	platelet derived growth factor C	1.9 (9.1)	2.55 (2.75)	2.63 (3.84)	6.04 (5.54)	2.86 (3.22)	7.74 (3.45)	—	0
*PIK3CB *	phosphatidylinositol-4,5-bisphosphate 3-kinase, catalytic subunit beta	—	1.96 (3.54)	2.8 (6.61)	2.57 (6.07)	2.08 (8.43)	3.55 (3.7)	2.99 (3.7)	3
*SOS2 *	son of sevenless homolog 2 (Drosophila)	1.58 (4.52)	2.32 (4.43)	2.66 (5.3)	2.41 (4.77)	2.67 (7.69)	3.57 (3.7)	—	0
*GSN *	gelsolin	1.87 (10.35)	2.09 (4.28)	1.63 (3.04)	1.59 (2.47)	2.09 (4.99)	2.93 (3.74)	—	2
*SSH1 *	slingshot homolog 1	1.67 (4.24)	2.17 (4.97)	2.12 (4.54)	1.68 (3.17)	1.69 (2.17)	4.85 (2.4)	—	0
*FGF13 *	fibroblast growth factor 13	—	3.6 (3.81)	4.87 (5.35)	6.27 (10.06)	12.36 (4.07)	105.9 (2.54)	—	0
*DIAPH2 *	diaphanous homolog 2	—	1.65 (2.4)	2.23 (6.84)	2.47 (7.12)	2.08 (4.64)	2.71 (2.21)	—	0
*PAK2 *	p21 (CDKN1A)-activated kinase 2	—	1.76 (4.62)	1.82 (5.18)	—	1.88 (9.03)	1.70 (3.78)	1.84 (2.27)	0
*CD14 *	CD14 molecule	1.54 (5.97)	2.51 (4.48)	2.33 (3.18)	—	1.54 (2.56)	1.77 (2)	—	1564
*ITGA2B *	integrin, alpha 2b (platelet glycoprotein IIb of IIb/IIIa complex, antigen CD41)	3.09 (9.06)	1.74 (2.6)	—	4.42 (4.9)	—	3.44 (3.84)	—	0
*BRAF *	B-Raf proto-oncogene, serine/threonine kinase	—	1.94 (3.7)	2.2 (9.68)	2.68 (8.92)	—	1.90 (2.04)	—	2
*MYL9 *	myosin, light chain 9, regulatory	3.39 (10.79)	—	—	3.38 (3.84)	2.25 (2.45)	2.53 (2.01)	—	0
*MAPK1 *	mitogen-activated protein kinase 1	—	2 (7.85)	1.85 (2.74)	1.79 (6.61)	—	1.89 (4.94)	—	137

GEO series #1 to 7 are listed: GSE30119, GSE8121, GSE9692, GSE28750, GSE13015-6106, GSE13015-6947 and GSE21802.

^∗^Number of publications associated with search terms “sepsis”, “severe sepsis” or “septic shock” in PubMed by searching the PubMatrix database.

## Abbreviations

LPS: Lipopolysaccharide
GEO: Gene Expression Omnibus
CLP: Cecal ligation and puncture
DAVID: Database for Annotation, Visualization, and Integrated Discovery
MSI: Mean signal intensity
SAM: Significance Analysis of Microarray
FDRs: False discovery rates
KEGG: Kyoto Encyclopedia of Genes and Genomes
PBMCs: Peripheral blood mononuclear cells
HMVECs: Human lung microvascular endothelial cells.
