# Supplementary material for: Lysosome and Cytoskeleton Pathways Are Robustly Enriched in the Blood of Septic Patients: A Meta-Analysis of Transcriptomic Data
Source: Mediators Inflamm. 2015 Apr 30;2015:984825. doi: 10.1155/2015/984825 (PMC4430672; doi:10.1155/2015/984825)
Supplement: Supplementary file 1 — The Supplementary Material includes one supplementary table and two additional supplementary figures that are complementary to the results reported in the final paper. These data are peer-reviewed and are subject to the same criteria as data in the main article. Each of the two figures is displayed with their legends placed below each corresponding figure [file 984825.f1.pdf]

Lysosome and Cytoskeleton Pathways are Robustly Enriched in the Blood of Septic Patients: A Meta-Analysis of Transcriptomic Data

Jie Ma[1,2], Chuanxi Chen[1,2,*], Andreas S. Barth[3], Chris Cheadle[1], Xiangdong Guan[2**], Li Gao[1**]

Data Supplement

Supplement Table S1. Processing of samples of included studies

GEO accession	Total RNA	Microarray hybridization	Normalization
GSE30119	Isolated from whole blood	Standard Illumina hybridization protocol	Average normalization with Genome Studio
GSE8121	Isolated from whole blood	Standard Affymetrix hybridization [illegible]	RMA normalization using the GeneSpring 7.2
GSE9692	Isolated from whole blood	Standard Affymetrix hybridization	RMA normalization using the GeneSpring GX 7.1
GSE28750	Isolated from whole blood	HGU133 Pllus 2.0 Genechips	A combination of Affymetrix Power Tools, R and Perl scripts to filter background noise and normalize data
GSE13015* Platform6106	Isolated from whole blood	Sentrix Human-6 v2 Expression BeadChip	BeadStudio version 1.5.1.3 using the average chip normalization method
GSE13015* Platform6947	Isolated from whole blood	HumanHT-12 Expression BeadChip	BeadStudio version 3.1.3.0 using the average chip normalization method
GSE21802	Isolated from whole blood	Standard Illumina hybridization protocol	Quantile normalization with Illumina GenomeStudio_2010.1

Supplement Figure 1. KEGG pathways involved in human sepsis. 199 (out of a total of 429) KEGG pathways were involved in human sepsis and visualized by Genesis software.

Supplement Figure 2. Graphical representations of protein interactions enriched for drug targets. We queried the Unified Human Interactome database (UniHI 7) with a list of top 15 transcripts from each of the two pathways (orange and blue for lysosome and cytoskeleton, respectively). The display was restricted to those interactions (yellow proteins) supported by at least three PubMed references. Molecular network analysis revealed that 22 out of 30 transcripts formed a network with multiple interconnected nodes (hubs) between top genes in both pathways. Importantly, many of the predicted interaction partners (red proteins) were drug targets.

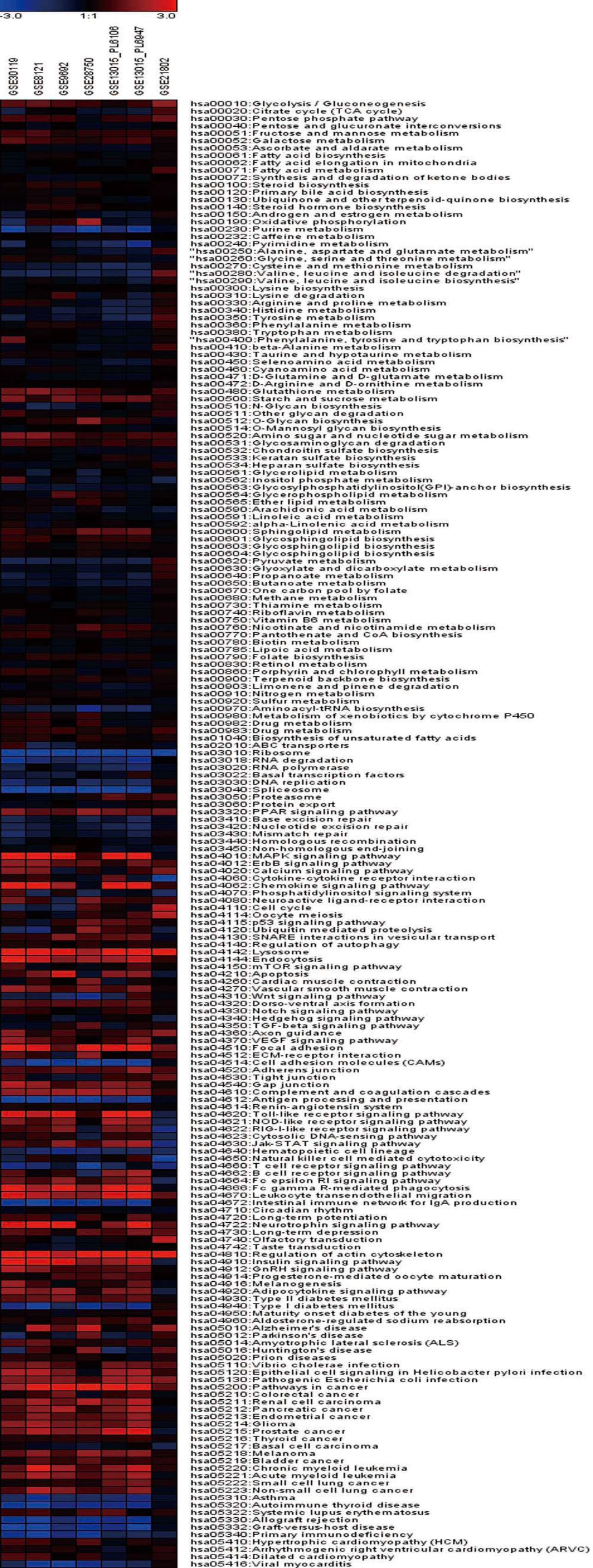
-3.0
1:1
3.0
GSE30119
GSE8121
GSE9692
GSE28750
GSE13015_PL6106
GSE13015_PL6947
GSE21802
hsa00010:Glycolysis / Gluconeogenesis
hsa00020:Citrate cycle (TCA cycle)
hsa00030:Pentose phosphate pathway
hsa00040:Pentose and glucuronate interconversions
hsa00051:Fructose and mannose metabolism
hsa00052:Galactose metabolism
hsa00053:Ascorbate and aldarate metabolism
hsa00061:Fatty acid biosynthesis
hsa00062:Fatty acid elongation in mitochondria
hsa00071:Fatty acid metabolism
hsa00072:Synthesis and degradation of ketone bodies
hsa00100:Steroid biosynthesis
hsa00120:Primary bile acid biosynthesis
hsa00130:Ubiquinone and other terpenoid-quinone biosynthesis
hsa00140:Steroid hormone biosynthesis
hsa00150:Androgen and estrogen metabolism
hsa00190:Oxidative phosphorylation
hsa00230:Purine metabolism
hsa00232:Caffeine metabolism
hsa00240:Pyrimidine metabolism
"hsa00250:Alanine, aspartate and glutamate metabolism"
"hsa00260:Glycine, serine and threonine metabolism"
hsa00270:Cysteine and methionine metabolism
"hsa00280:Valine, leucine and isoleucine degradation"
"hsa00290:Valine, leucine and isoleucine biosynthesis"
hsa00300:Lysine biosynthesis
hsa00310:Lysine degradation
hsa00330:Arginine and proline metabolism
hsa00340:Histidine metabolism
hsa00350:Tyrosine metabolism
hsa00360:Phenylalanine metabolism
hsa00380:Tryptophan metabolism
"hsa00400:Phenylalanine, tyrosine and tryptophan biosynthesis"
hsa00410:beta-Alanine metabolism
hsa00430:Taurine and hypotaurine metabolism
hsa00450:Selenoamino acid metabolism
hsa00460:Cyanoamino acid metabolism
hsa00471:D-Glutamine and D-glutamate metabolism
hsa00472:D-Arginine and D-ornithine metabolism
hsa00480:Glutathione metabolism
hsa00500:Starch and sucrose metabolism
hsa00510:N-Glycan biosynthesis
hsa00511:Other glycan degradation
hsa00512:O-Glycan biosynthesis
hsa00514:O-Mannosyl glycan biosynthesis
hsa00520:Amino sugar and nucleotide sugar metabolism
hsa00531:Glycosaminoglycan degradation
hsa00532:Chondroitin sulfate biosynthesis
hsa00533:Keratan sulfate biosynthesis
hsa00534:Heparan sulfate biosynthesis
hsa00561:Glycerolipid metabolism
hsa00562:Inositol phosphate metabolism
hsa00563:Glycosylphosphatidylinositol(GPI)-anchor biosynthesis
hsa00564:Glycerophospholipid metabolism
hsa00565:Ether lipid metabolism
hsa00590:Arachidonic acid metabolism
hsa00591:Linoleic acid metabolism
hsa00592:alpha-Linolenic acid metabolism
hsa00600:Sphingolipid metabolism
hsa00601:Glycosphingolipid biosynthesis
hsa00603:Glycosphingolipid biosynthesis
hsa00604:Glycosphingolipid biosynthesis
hsa00620:Pyruvate metabolism
hsa00630:Glyoxylate and dicarboxylate metabolism
hsa00640:Propanoate metabolism
hsa00650:Butanoate metabolism
hsa00670:One carbon pool by folate
hsa00680:Methane metabolism
hsa00730:Thiamine metabolism
hsa00740:Riboflavin metabolism
hsa00750:Vitamin B6 metabolism
hsa00760:Nicotinate and nicotinamide metabolism
hsa00770:Pantothenate and CoA biosynthesis
hsa00780:Biotin metabolism
hsa00785:Lipoic acid metabolism
hsa00790:Folate biosynthesis
hsa00830:Retinol metabolism
hsa00860:Porphyrin and chlorophyll metabolism
hsa00900:Terpenoid backbone biosynthesis
hsa00903:Limonene and pinene degradation
hsa00910:Nitrogen metabolism
hsa00920:Sulfur metabolism
hsa00970:Aminoacyl-tRNA biosynthesis
hsa00980:Metabolism of xenobiotics by cytochrome P450
hsa00982:Drug metabolism
hsa00983:Drug metabolism
hsa01040:Biosynthesis of unsaturated fatty acids
hsa02010:ABC transporters
hsa03010:Ribosome
hsa03018:RNA degradation
hsa03020:RNA polymerase
hsa03022:Basal transcription factors
hsa03030:DNA replication
hsa03040:Spliceosome
hsa03050:Proteasome
hsa03060:Protein export
hsa03320:PPAR signaling pathway
hsa03410:Base excision repair
hsa03420:Nucleotide excision repair
hsa03430:Mismatch repair
hsa03440:Homologous recombination
hsa03450:Non-homologous end-joining
hsa04010:MAPK signaling pathway
hsa04012:ErbB signaling pathway
hsa04020:Calcium signaling pathway
hsa04060:Cytokine-cytokine receptor interaction
hsa04062:Chemokine signaling pathway
hsa04070:Phosphatidylinositol signaling system
hsa04080:Neuroactive ligand-receptor interaction
hsa04110:Cell cycle
hsa04114:Oocyte meiosis
hsa04115:p53 signaling pathway
hsa04120:Ubiquitin mediated proteolysis
hsa04130:SNARE interactions in vesicular transport
hsa04140:Regulation of autophagy
hsa04142:Lysosome
hsa04144:Endocytosis
hsa04150:mTOR signaling pathway
hsa04210:Apoptosis
hsa04260:Cardiac muscle contraction
hsa04270:Vascular smooth muscle contraction
hsa04310:Wnt signaling pathway
hsa04320:Dorso-ventral axis formation
hsa04330:Notch signaling pathway
hsa04340:Hedgehog signaling pathway
hsa04350:TGF-beta signaling pathway
hsa04360:Axon guidance
hsa04370:VEGF signaling pathway
hsa04510:Focal adhesion
hsa04512:ECM-receptor interaction
hsa04514:Cell adhesion molecules (CAMs)
hsa04520:Adherens junction
hsa04530:Tight junction
hsa04540:Gap junction
hsa04610:Complement and coagulation cascades
hsa04612:Antigen processing and presentation
hsa04614:Renin-angiotensin system
hsa04620:Toll-like receptor signaling pathway
hsa04621:NOD-like receptor signaling pathway
hsa04622:RIG-I-like receptor signaling pathway
hsa04623:Cytosolic DNA-sensing pathway
hsa04630:Jak-STAT signaling pathway
hsa04640:Hematopoietic cell lineage
hsa04650:Natural killer cell mediated cytotoxicity
hsa04660:T cell receptor signaling pathway
hsa04662:B cell receptor signaling pathway
hsa04664:Fc epsilon RI signaling pathway
hsa04666:Fc gamma R-mediated phagocytosis
hsa04670:Leukocyte transendothelial migration
hsa04672:Intestinal immune network for IgA production
hsa04710:Circadian rhythm
hsa04720:Long-term potentiation
hsa04722:Neurotrophin signaling pathway
hsa04730:Long-term depression
hsa04740:Olfactory transduction
hsa04742:Taste transduction
hsa04810:Regulation of actin cytoskeleton
hsa04910:Insulin signaling pathway
hsa04912:GnRH signaling pathway
hsa04914:Progesterone-mediated oocyte maturation
hsa04916:Melanogenesis
hsa04920:Adipocytokine signaling pathway
hsa04930:Type II diabetes mellitus
hsa04940:Type I diabetes mellitus
hsa04950:Maturity onset diabetes of the young
hsa04960:Aldosterone-regulated sodium reabsorption
hsa05010:Alzheimer's disease
hsa05012:Parkinson's disease
hsa05014:Amyotrophic lateral sclerosis (ALS)
hsa05016:Huntington's disease
hsa05020:Prion diseases
hsa05110:Vibrio cholerae infection
hsa05120:Epithelial cell signaling in Helicobacter pylori infection
hsa05130:Pathogenic Escherichia coli infection
hsa05200:Pathways in cancer
hsa05210:Colorectal cancer
hsa05211:Renal cell carcinoma
hsa05212:Pancreatic cancer
hsa05213:Endometrial cancer
hsa05214:Glioma
hsa05215:Prostate cancer
hsa05216:Thyroid cancer
hsa05217:Basal cell carcinoma
hsa05218:Melanoma
hsa05219:Bladder cancer
hsa05220:Chronic myeloid leukemia
hsa05221:Acute myeloid leukemia
hsa05222:Small cell lung cancer
hsa05223:Non-small cell lung cancer
hsa05310:Asthma
hsa05320:Autoimmune thyroid disease
hsa05322:Systemic lupus erythematosus
hsa05330:Allograft rejection
hsa05332:Graft-versus-host disease
hsa05340:Primary immunodeficiency
hsa05410:Hypertrophic cardiomyopathy (HCM)
hsa05412:Arrhythmogenic right ventricular cardiomyopathy (ARVC)
hsa05414:Dilated cardiomyopathy
hsa05416:Viral myocarditis

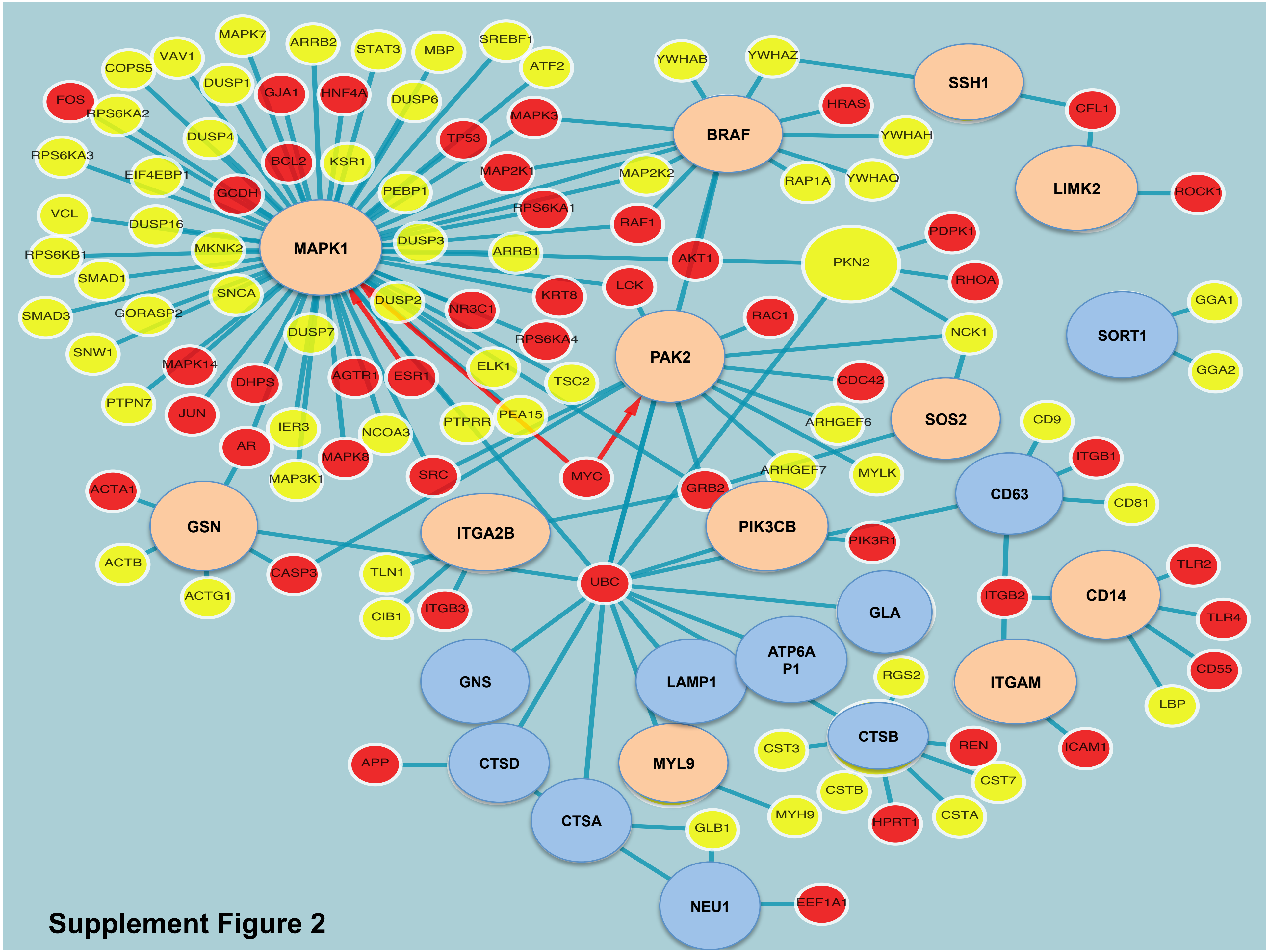

Supplement Figure 2
